# The long road to recovery: at six months since the first COVID-19 wave, elective orthopedic care has still not fully recovered in Belgium

**DOI:** 10.1186/s40634-020-00316-9

**Published:** 2020-12-21

**Authors:** Y. N. Tan, PJ. Vandekerckhove, P Verdonk

**Affiliations:** 1 Department of Orthopedics and Traumatology, University Hospital Brussels, Laarbeeklaan 101, 1090 Jette, Belgium; 2 Department of Orthopedics and Traumatology, Sint-Jan Hospital, Ruddershove 10, 8000 Orthoclinic, AZ Belgium; 3 ORTHOCA, AZ Monica, Stevenslei 20, 2100 Deurne, Belgium; 4 Department of Orthopedics and Traumatology, University Hospital Antwerp, Antwerp, Belgium

**Keywords:** COVID-19, Orthopaedic surgery, Knee surgery, Outpatient visits, Elective care, Safety

## Abstract

**Purpose:**

The primary aim of our study was to investigate elective orthopaedic care during the first wave government-imposed COVID-19 lockdown and at four weeks and 21 weeks after resuming elective care. The secondary aim of our study was to evaluate the implementation of the European Society of Sports Traumatology, Knee Surgery and Arthroscopy (ESSKA) COVID-19 Guidelines and Recommendations for Resuming Elective Surgery in the clinical practice of Belgian knee surgeons.

**Methods:**

We sent three anonymous online surveys to 102 Belgian Knee Society members (BKS) at times mentioned above. Addressed topics were: (1) participant demographics, (2) elective surgeries, (3) outpatient visits, (4) ESSKA Guidelines, (5) patient and surgeon safety.

**Results:**

During the COVID-19 lockdown, there was a decrease of 97% in elective knee surgeries and 91% in outpatient visits. At four and 21 weeks after resuming elective care, volumes were respectively 67% and 89% for elective surgeries and 81% and 91% for outpatient visits. Regarding ESSKA guidelines, 91% of surgeons had no COVID-19 testing prior to resuming elective care. Ninety-two per cent reported preoperative (< 72 h) patient PCR testing, and 45% gave preference to young patients without comorbidities. Seventy-two per cent did not use additional personal protective equipment (PPE) if a patient PCR test was negative. Forty-nine per cent continued to give preference to general anaesthesia.

**Conclusion:**

Our study shows that elective surgeries and outpatient visits were almost completely interrupted during the COVID-19 lockdown and were still below normal at four and 21 weeks after resuming elective care. Regarding ESSKA COVID-19 guidelines, our study observes good compliance in preoperative patient COVID-19 testing, but lower compliance for preoperative health care personnel testing, patient selection, use of PPE, and locoregional anaesthesia.

**Level of Evidence:**

V

**Supplementary Information:**

The online version contains supplementary material available at 10.1186/s40634-020-00316-9.

## Introduction

The COVID-19 outbreak has impacted all health care systems across Europe. In March 2020, many governments across Europe ordered a shutdown on elective surgeries and regular non-urgent outpatient visits, including primary and revision knee arthroplasty and other non-urgent orthopaedic care [2, 9, 15].

Since May 2020, most European countries slowly restarted elective activities, as new COVID-19 cases and intensive care unit (ICU) admissions gradually reduced.

At the same time, multiple guidelines and recommendations have been developed to aid orthopaedic surgeons to safely restart activities after the first wave COVID-19 lockdown [3, 7, 12, 13].

Data on the recovery of elective orthopaedic care after the first COVID-19 wave has remained sparse; Liebensteiner et al. conducted a survey directly after the COVID-19 first wave peak, ﻿reporting that approximately 90% of surgeons experienced substantial reductions in surgical caseload and patient contact [10].

As many European orthopaedic departments are currently facing new uncertainties as a result of a second COVID-19 wave, the collection of data on orthopaedic performance is particularly important [4].

The primary aim of this study was to provide the orthopaedic community with data on the recovery of elective care. We do so by investigating the resumption of elective care in the first six months after the first wave COVID-19 lockdown by comparing the amount of elective knee surgeries and outpatient clinic visits during: (1) the COVID-19 lockdown, (2) the four weeks after restart of elective care, (3) at 21 weeks after resuming elective care (corresponding to six months after the introduction of the first wave COVID-19 lockdown), and (4) before the COVID-19 crisis.

The secondary aim of our study was to evaluate the implementation of the ESSKA COVID-19 Guidelines and Recommendations for Resuming Elective Surgery in the clinical practice of Belgian knee surgeons after the restart of elective care [12].

## Material and methods

We performed three online surveys among members of the Belgian Knee Society (BKS). The BKS is a recognized subgroup of the Belgian Association of Orthopedics and Traumatology and consists of 102 knee surgeons in the Flemish area of Belgium.

The first survey focused on orthopaedic activities during the seven-week government-imposed lockdown which started on 15 March 2020 and lasted until 1 May 2020 and was sent to BKS members on 1 May 2020.

The second survey focused on evaluating the first four weeks of the restart of elective surgeries and outpatient visits (starting on 4 May and ending on 31 May 2020) and was sent to BKS members on 7 June 2020.

A third survey focused on elective surgeries and outpatient visits at 21 weeks after resuming elective care (14 until 21 September 2020), which corresponds to six months after the introduction of the first wave COVID-19 lockdown measures. The survey was sent to BKS members on 21 September 2020.

The first survey consisted of 17 questions and addressed three main topics: (1) participant demographics and experience, (2) elective surgery related questions, and (3) outpatient visit related questions. To better understand the impact of the COVID-19 lockdown, additional questions were included with regard to orthopaedic activity during the same period last year (15 March until 1 May 2019) (Additional file 1). The second survey consisted of 36 questions and addressed four main topics: (1) participant demographics and experience, (2) elective surgery related questions, (3) outpatient visit related questions, and (4) implementation of the ESSKA COVID-19 Guidelines and Recommendations related questions (Additional file 2). The third survey consisted of 17 questions and addressed four main topics: (1) patient demographics, (2) elective surgery related questions, (3) outpatient related questions, and (4) patient and surgeon safety related questions (Additional file 3).

No approval was obtained from an institutional review board because the survey was anonymous, and no patient data were included. The surveys were conducted using the online data collection platform www.surveymonkey.com. A direct link was sent by email to the members, and participation of the survey was limited to one time per email address. For all surveys, the members were given one week to respond, and a reminder to complete the survey was sent to the members two days before the deadline. The responses were analyzed for frequencies, means, and standard deviations (SD) using SPSS.

Due to the lack of national guidelines for orthopaedic surgeons, the ESSKA COVID-19 Guidelines and Recommendations for Resuming Elective Surgery were communicated to Belgian knee surgeons as being an interesting tool to restart elective surgery and were at no point mandatory. They were communicated because of their clear and practical guidelines and ESSKA’s specific expertise in knee surgery.

The guidelines and recommendations consist of four main sections. Section 1 describes preoperative management and recommends a COVID-19 free facility, (weekly) testing of all health care personnel, patient selection based on age, COVID-19 exposure, ASA scores, socio-professional situation, and preoperative patient screening (preoperative questionnaire, PCR and/or immune serology test < 72 h prior to surgery, preoperative blood test, vital signs monitoring on the day of surgery, and monitoring COVID-19 symptoms at arrival in the hospital). Section 2 describes surgical indications and gives, among others, a recommendation on the type of surgery (emergency, urgent, somewhat elective, elective) and the preference for locoregional anaesthesia. Section 3 gives a recommendation on personal protective equipment (PPE) for the orthopaedic and trauma surgeon. This includes, among others, the use of level four surgical gowns, FFP2 masks, and eye protection during surgery. It also gives an overview of high aerosol-generating procedures (AGP’s), such as electrocautery, drill, jet lavage systems, and power tools. Section 4 gives a recommendation on the postoperative care, recommends an introduction of video consultations and access to adequate postoperative care, such as physiotherapy before resuming elective surgeries. [12]

Due to the extensiveness of the guidelines, three members of the BKS board chose items of the guidelines, which were thought of as being most relevant and controllable by orthopaedic surgeons.

## Results

### Participants demographics

A total of 54 (53%) knee surgeons participated in the first survey, 59 (58%) participated in the second survey, and 56 (54%) participated in the third survey. Fifty-five per cent of the participants had more than 20 years of experience as an orthopaedic surgeon. Ninety per cent of participants were working in non-academic hospitals.

### Impact of the first wave COVID-19 lockdown on orthopaedic practice

**Fig. 1:**
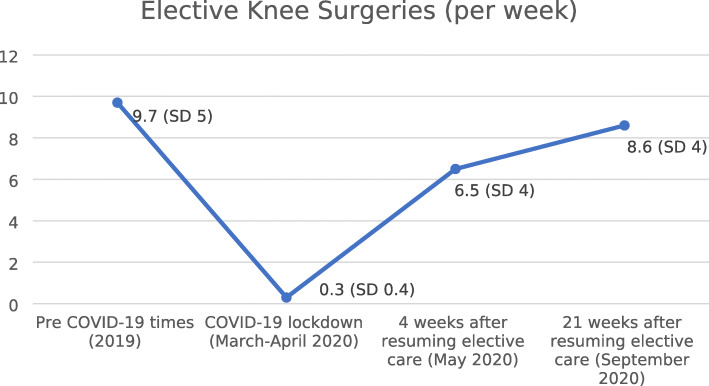
Amount of elective knee surgeries per week during: pre-COVID-19 times (March- April 2019); COVID-19 lockdown (March–April 2020); 4 weeks after resuming elective orthopaedic care (May 2020); 21 weeks after resuming elective care (September 2020; corresponding to 6 months after the introduction of first wave COVID-19 lockdown)

When comparing the elective surgeries during the seven-week lockdown period to the same period in 2019, there was a decrease of 97% in mean surgeries from 9.7 (SD 5) surgeries per week in 2019, compared to 0.3 (SD 0.4) per week during the lockdown period (Fig. 1). A total of 125 knee surgeries among the participants were reported during the lockdown period. The most frequently performed surgery was meniscectomy (38%) followed by anterior cruciate ligament reconstruction (24%), meniscus repair (14%), septic revision TKA (7%), and primary knee arthroplasty (5%).

**Fig. 2:**
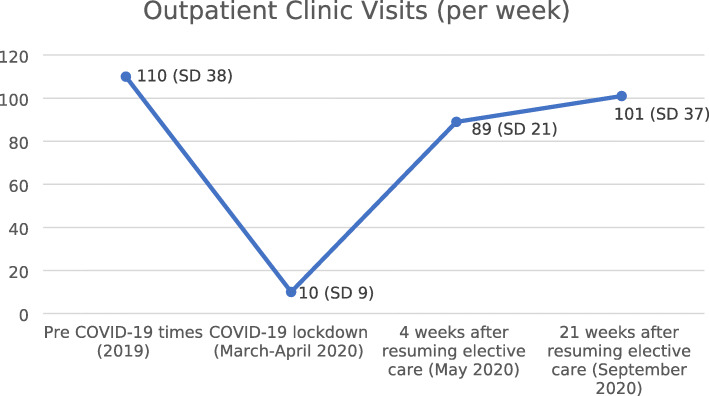
Amount of outpatient clinic visits per week during: pre-COVID-19 times (March—April 2019); COVID-19 lockdown (March–April 2020); 4 weeks after resuming elective orthopaedic care (May 2020); 21 weeks after resuming elective care (September 2020; corresponding to 6 months after the introduction of first wave COVID-19 lockdown)

During the same period, the mean number of outpatient visits decreased 91%, from 110 (SD 38) per week in 2019 to 10 (SD 9) per week in 2020 (Fig. 2).

At the same time, 93% of participants conducted video consultations during the lockdown period with a mean of 9 (SD 11.8) consultations per week.

### Elective orthopaedic surgery and regular outpatient clinic visits in the first month of the restart of elective care

Starting 4 May 2020, the government gave permission to restart elective surgeries and outpatient visits.

In the first week after resuming elective care, 89% of the participants reported having access to 50% or less operation room (OR) time. In the fourth week after resuming elective care, the available OR time increased; however, only 14% of participants had 100% OR time compared to pre-COVID-19 lockdown times.

The mean number of elective surgical procedures during the first month after lockdown decreased to 67% from 9.7 (SD 5) per week in 2019 to 6.5 (SD 4) per week during the same period in 2020 (Fig. 1).

Almost 60% of participants experienced an increase in waiting time for surgery by four weeks (mean waiting time increased from five to nine weeks). The main reasons given for this reported increase are a lack of OR personnel due to back up for ICU (58%), lack of personnel in general (40%), lack of hospital beds (37%), and lack of goodwill of hospital management (26%).

Twenty per cent of participants reported a decrease in the duration of hospital stay for primary total knee arthroplasty (TKA) (mean of 3.8 days to 2.7 days).

During the first week of the restart, the participants saw a mean of 68 (SD 17) patients at the outpatient clinic compared to a mean of 110 (SD 38) per week prior to the COVID-19 lockdown. In week four, after the restart, the mean was 89 (SD 21) or 81% compared to non-COVID-19 times (Fig. 2).

Fifty-nine per cent of the participants reported a decrease in demand for outpatient visits compared to non-COVID-19 times. Thirty-four per cent reported the same demand, and only 7% reported an increased demand for outpatient visits.

At one month after the restart for outpatient visits, 41% of the participants reported to no longer use video consultations, 37% was still using video consultations but would prefer to stop using it when the COVID-19 crisis comes to an end, and 22% would like to continue using it regardless of the COVID-19 crisis.

### Comparing Belgian practice to the ‘ESSKA COVID-19 guidelines and recommendations for resuming elective surgery’

**Fig. 3:**
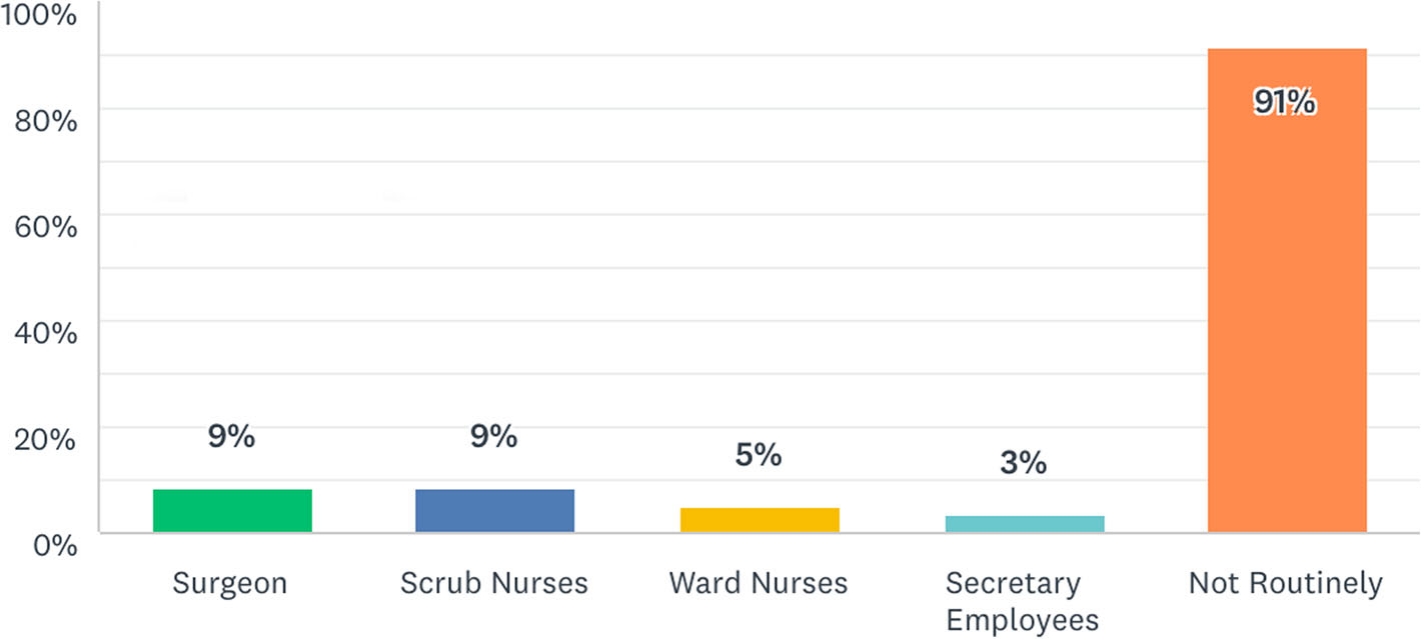
Percentage of COVID-19 testing (PCR or immune testing) among health care personal at the time of resuming elective orthopaedic care (4rd May 2020)

At the restart of elective care, 91% of participants had no COVID-19 testing of any kind for health care personnel. Nine per cent reported COVID-19 PCR testing for surgeons and the OR team, 5% reported COVID-19 PCR testing of surgical ward nurses, and 3% reported testing of secretary employees (Fig. 3).

Thirty-six per cent of the participants gave a specific preference for short-stay procedures (day care or one-night stay), in contrast to 59% of the participants who had no specific preference and restarted with all types of knee surgery. Five per cent had a preference for procedures requiring a longer hospitalization stay, such as total knee arthroplasty.

Regarding patient selection, 45% gave a preference for younger patient or patients without important comorbidities.

**Fig. 4:**
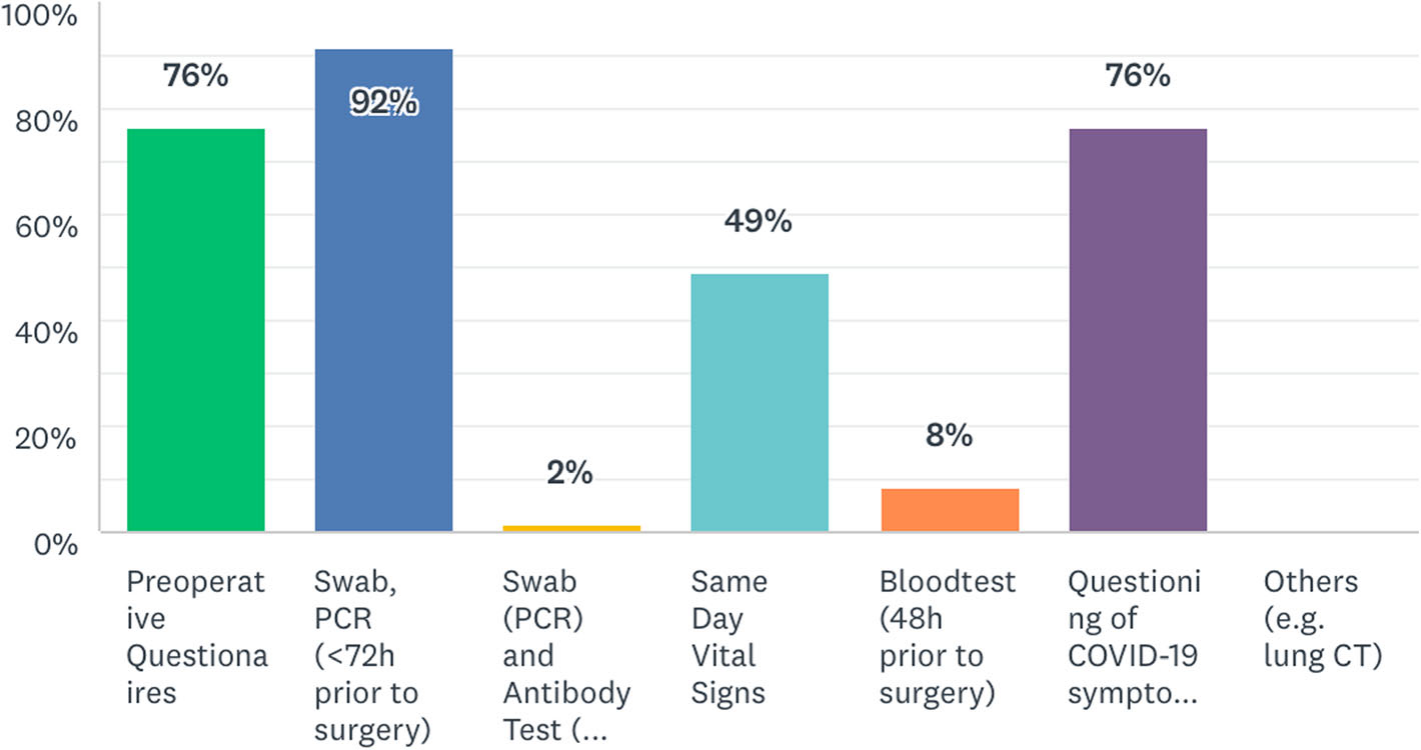
Preoperative patient testing prior to elective surgery (preoperative COVID-19 questionnaires; COVID-19 swab PCR test < 72 h prior to surgery; COVID-19 swab PCR test and antibody testing; same-day vital signs monitoring; blood test for inflammatory signs < 48 h prior to surgery; questioning of COVID-19 symptoms on same day of surgery; others)

When it comes to preoperative patient COVID-19 testing, 92% performed PCR testing < 72 h before surgery. Additional screening measures were preoperative COVID-19 symptom questionnaires (76%), questioning of symptoms on the same day (76%), measurement of vital parameters at admission on the day of surgery (49%), blood test for inflammatory signs < 48 h prior to surgery (8%), and PCR and antibody testing (2%) (Fig. 4).

Regarding PPE, 72% of the surgeons reported not taking any additional precautions compared to non-COVID-19 times if the patient had a negative PCR test. Twenty-six per cent of the participants wore protective eyewear more often, and 8% wore a FFP2 mask at all times during surgery.

During surgery, 93% said they did not perform technical adaptations to control aerosol-generating procedures (AGP’s), such as a decreased usage of electrocautery, high-speed power tools, or jet lavage systems*.* There was also no increase in the use of exsanguination or tourniquet.

**Fig. 5:**
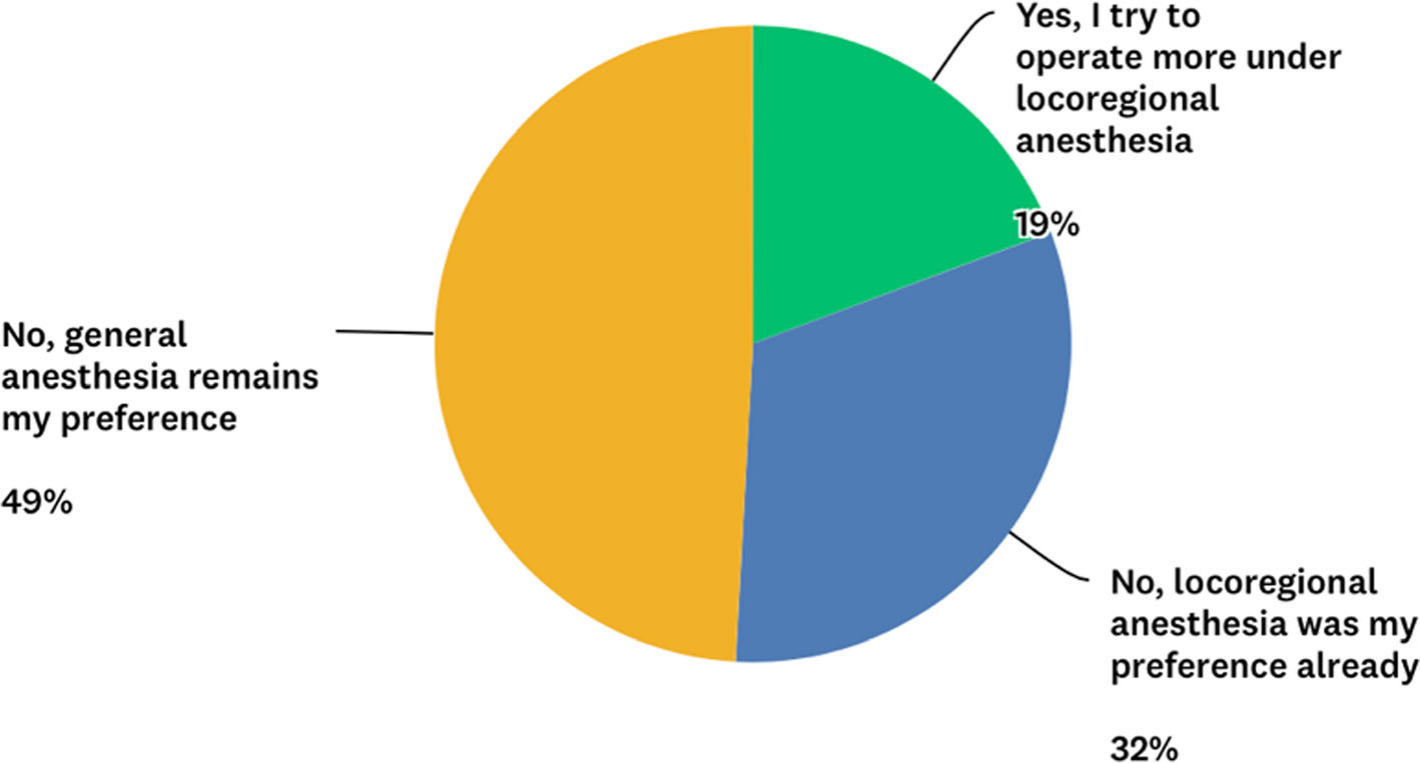
Preference of type of anaesthesia (general vs locoregional anaesthesia) for elective care during the COVID-19 pandemic

With regard to the preference of anaesthesia, 49% of the participants continued to give have preference for general anaesthesia over locoregional anaesthesia. Nineteen per cent reported having a preference for locoregional anaesthesia as a consequence of the COVID-19 crisis, and 32% already had a preference for locoregional anaesthesia (Fig. 5).

### Orthopaedic knee care at 21 weeks after the restart of elective orthopaedic care

At 21 weeks after the restart of elective orthopaedic care (corresponding to six months after the introduction of the COVID-19 lockdown measures), the participants reported a mean of 8.6 (SD 5) surgeries per week, or 89% compared to pre-COVID-19 (Fig. 1). However, not all surgeons performed fewer surgical procedures: 7% reported more surgical procedures, and 43% reported the same amount of surgical procedures compared to pre-COVID-19 times. The remaining 47% of knee surgeons reported having performed fewer surgical procedures compared to pre-COVID-19 times. Similar to the decrease in surgical procedures, the available OR time decreased to a mean of 90% compared to pre-COVID-19 times.

Regarding outpatient visits, participants reported a mean of 101 (SD 38) visits per week, or 91% compared to pre-COVID-19 times (Fig. 2). Fifty per cent continued to report a smaller demand for outpatient visits, 41% reported the same demand, and 9% reported a greater demand compared to pre-COVID-19 times. Only 25% of participants were still using video consultations (compared to 97% during the COVID-19 lockdown, and 59% at one month after the restart for elective care). For those still using video consultations, the mean number continued to decrease from nine (SD 11.8) per week during the COVID-19 lockdown, to two (SD 5) per week at 21 weeks after resuming elective care.

### Surgeon and patient safety in the first six months since the start of the COVID-19 lockdown

During the first six months (16 March – 21 September 2020) of the COVID-19 crisis, 3% of knee surgeons had a positive PCR COVID-19 test. Ninety per cent of surgeons had no known COVID-19 symptoms, or had a negative COVID-19 PCR test. Seven per cent did not have a positive COVID-19 PCR test but did have one or more of COVID-19 symptoms. During this period, a mean of 2.6 (SD 5.1) of elective knee surgeries had to be cancelled or postponed due to a preoperative positive patient COVID-19 PCR test. Regarding patient safety, since the restart of elective care, none of the participants reported a known patient COVID-19 infection during a hospital stay for an elective procedure. There was also no postoperative COVID-19 infection which could be traced back to the elective procedure hospital stay. Regarding outpatient visits, none of the participants reported a patient COVID-19 infection that could be traced back to the hospital visit.

## Discussion

The most important finding of the present study is that elective surgeries and outpatient visits were almost completely interrupted during the seven-week government-imposed first wave COVID-19 lockdown and were still largely below normal numbers at four and 21 weeks after resuming elective care.

A second important finding is that despite not reaching complete ESSKA guidelines compliance, the restart of elective surgery and outpatient visit was safe.

Up until now, our study is the first to show a continued delay or postponement of orthopaedic care even at six months after the initiation of the first wave COVID-19 shutdown. Liebensteiner et al. reported severe disruptions of orthopaedic activity shortly after the first COVID-19 wave in 90% of their correspondents [10]. Jain et al. described prediction models claiming that it would take seven months in the most optimistic scenario until the orthopaedic health-care system could perform 90% of the expected pre-pandemic forecasted volume of surgeries [6]. This correlates with our findings of 89% of elective surgery volumes and 91% of outpatient visits at six months after the initiation of the COVID-19 shutdown, compared to pre-pandemic volumes.

When comparing our data to the ESSKA COVID-19 guidelines and recommendations, our study observed a mixed compliance. We observed a good score in preoperative patient COVID-19 testing but observed lower scores for preoperative health care personnel testing, patient selection, use of PPE, and locoregional anaesthesia.

One of the difficult choices orthopaedic surgeons face is which patients to prioritize. The ESSKA guidelines, the international consensus group and the AAHKS research group, recommended prioritizing younger patients, or patients without comorbidities [13, 14]. As it is likely that the COVID-19 pandemic will not resolve soon, older patients might see their arthroplasty postponed for more than a year. The adverse impact of arthroplasty postponement on patient mobility, opioid consumption, and mental health have already been studied [1, 5, 11]. Therefore, careful consideration should be made to not exclude these patients from orthopaedic care during the pandemic. In our study, 45% of surgeons gave preference to young patients without comorbidities.

While the guidelines and recommendations offer a useful tool for clinicians, in reality, a complete compliance to the guidelines seems to be challenging. It requires a strong collaboration and commitment of many stakeholders (national and local government, hospital management, health care personnel, and interdisciplinary cooperation), infrastructure (COVID-19 free institution), sufficient financial means, and medical equipment availability. Recently the EHS and EKA published more extensive recommendations for resuming elective care, which included many references to the ESSKA guidelines [7, 8].

Despite not reaching full ESSKA guidelines compliance, our study shows that safe surgery during the COVID-19 pandemic is possible. While Belgium has an extensive national COVID-19 contact tracing system, we observed no patient COVID-19 infection that could be traced back to an elective surgery hospitalization or outpatient visit. This observation is important in supporting the European Hip Society and European Knee associates goal to continue arthroplasty as long as possible during the COVID-19 pandemic [4].

Our study has several limitations. Firstly, the response rate of the three surveys was 53%, 58%, and 54%, respectively. Ideally, we would have preferred a higher response rate to have an even better representation of orthopaedic knee activities during the COVID-19 crisis. Another limitation is that our survey only included Belgian knee surgeons, and therefore these findings cannot be generalised to all European countries. Also, not all the sections of the ESSKA COVID-19 Guidelines and Recommendations were included in our survey. A more extended survey could have given a complete view of guidelines compliance.

The main strength of our paper is that we are the first to provide the orthopaedic community with data on the recovery of elective care up until six months after the first wave of the COVID-19 lockdown. Currently, many orthopaedic departments in Europe are experiencing new disruptions as a consequence of a second wave of COVID-19. We believe that our data on the recovery of elective orthopaedic care will be valuable in assisting orthopaedic departments with predicting and planning their activities after the current and potential future waves of COVID-19.

## Conclusion

Our study shows that elective surgeries and outpatient visits were almost completely interrupted during the COVID-19 lockdown and were still largely below normal numbers at four and 21 weeks after resuming elective care. Comparing clinical practice to the ESSKA COVID-19 Guidelines and Recommendations, our study shows a good compliance in preoperative patient COVID-19 testing, but observed lower compliance for preoperative health care personnel testing, patient selection, use of PPE, and locoregional anaesthesia*.* Despite not reaching full ESSKA guidelines compliance, our study did not observe a COVID-19 patient infection related to elective care, suggesting that safe elective orthopaedic care is possible during the COVID-19 pandemic and its aftermath. We believe that our findings are valuable to the orthopaedic community to better predict and plan elective activities during the current and potential future COVID-19 waves.

## Supplementary Information

**Additional file 1.** Overview of survey 1.

**Additional file 2.** Overview of survey 2.

**Additional file 3.** Overview of survey 3.

## Data Availability

The datasets used and/or analyzed during the current study are available from the corresponding author on reasonable request.
