# Supplementary material for: The long road to recovery: at six months since the first COVID-19 wave, elective orthopedic care has still not fully recovered in Belgium
Source: J Exp Orthop. 2020 Dec 21;7:99. doi: 10.1186/s40634-020-00316-9 (PMC7752098; doi:10.1186/s40634-020-00316-9)
Supplement: Supplementary file 1 — Additional file 1. Overview of survey 1. [file 40634_2020_316_MOESM1_ESM.docx]

Appendix 1

1. In which province are you working?

- Antwerp
- Limburg
- East Flanders
- West Flanders
- Flemish Brabant

2. How many percentage of your work is knee surgery?

- 25%

- 50%

- 75%

- 100%

3. How many years of experience do you have as an orthopedic surgeon?

- <5 years

- 5-10 years

- 10-20 years

- >20 years

4. How many elective knee surgeries did you perform from 15^th^ march until 1^st^ may 2019?

5. How many elective knee surgeries did you perform from 15^th^ march until 1^st^ may 2020?

6. How many of the following procedures did you perform from 15^th^ march until 1^st^ may 2020?

- Infection (Irrigation & Debridement, Spacer placement)

- Primary Arthroplasty

- Revision Arthroplasty

- ACL reconstruction

- MPFL reconstruction

- Acute primary ligament repair (MCL, MPFL, others)

- Menisectomy

- Meniscus repair

- Others

7. How many outpatient clinic visits did you perform from 15^th^ march until 1^st^ may 2019?

8. How many outpatient clinic visits did you perform from 15^th^ march until 1^st^ may 2020?

9. How many tele consultations did you perform from 15^th^ march until 1^st^ may 2020?

10. Will additional arrangements be made within your department/hospital in the coming months to clear the backlog in outpatient visits? (several answers possible)

- You are going to expand into the evening

- You are going to expand to the weekend

- No, you keep the same weekly work schedule

- You are going to extend the private practice

- You are going to take less holiday time

- Others

11. How many outpatient visits did you see on a weekly basis before this corona crisis?

12. How many outpatient visits will you be able to see per week taking into account COVID-19 measures in the first weeks after 4 May?

13. Can your elective surgery resume on 4 May? (multiple answers possible)

- Yes

- Yes, even earlier than 4rd May

- No still overload of ICU/ hospital beds

- No, Hospital orders

14. What percentage of OR capacity do you have available in May compared to the pre-corona crisis period?

- 0%

- 25%

- 50%

- 75%

- 100%

15. With which types of surgery will you resume again after the COVID-19 lockdown?

- Mainly one day surgery

- Mainly one day surgery and short stay hospitalizations (1 night) (meniscectomy, ACL reconstructions, osteotomy, ..)

- Mainly longer hospitalization stay procedure days (e.g. arthroplasty and revision surgery)

16. Do you give priority to certain patients during the surgical restart?

- Yes, preferably first younger patients (<60 years old) and minor surgery (no arthroplasty).

- Yes, preferably first younger patients (<60 years)

- No, I'm going to follow my previous waiting list

- No, preferably first patients >60 years

17. Following this Corona crisis, are you going to carry out more operations under locoregional anaesthesia?

- Yes, as much as possible

- Yes, but limited

- No, I prefer general anaesthesia
