# Supplementary material for: The long road to recovery: at six months since the first COVID-19 wave, elective orthopedic care has still not fully recovered in Belgium
Source: J Exp Orthop. 2020 Dec 21;7:99. doi: 10.1186/s40634-020-00316-9 (PMC7752098; doi:10.1186/s40634-020-00316-9)
Supplement: Supplementary file 2 — Additional file 2. Overview of survey 2. [file 40634_2020_316_MOESM2_ESM.docx]

Appendix 1

1. In which province are you working?

- Antwerp
- Limburg
- East Flanders
- West Flanders
- Flemish Brabant

2. How many percentage of your work is knee surgery

- 25%

- 50%

- 75%

- 100%

3. Where is your main working activity?

- University Hospital

- Non-University Hospital

4. What percentage of surgery did you start in the first week of May (when elective care resumed) compared to precorona times?

- <25%

- 25%

- 50%

- 75%

- 90%

- 100%

5. What percentage of surgery will you be at from 8 June compared to precorona times?

- <25%

- 25%

- 50%

- 75%

- 90%

- 100%

6. Has priority been given to other disciplines within your hospital?

- Yes

- Non, pro rata distribution of OR time between specialties

7. How many elective knee surgeries did you perform from 1^st^ may 2019 until 1^st^ June 2019 (pre-corona)?

8. How many elective knee surgeries did you perform 1^st^ may 2020 until 1^st^ June 2020?

9. What was the biggest limitation for the restart? (multiple options)

- Limited OR capacity (back up for ICU)

- Limited medical personnel

- Limited hospital admission capacity (hospital beds)

- Good will of hospital management

- Others

10. What interventions have you carried out from 1 May to 1 June 2020?

- Mainly same day discharge procedures (e.g. arthroscopy)

- Mainly same day and short-stay discharge procedures (e.g. HTO, ACL recon, ...)

- Mainly Long-stay (arthroplasty)

- All types of knee surgery

11. Have you given preference to certain types of patients?

- Yes, mainly young people / patients without comorbidity

- No

12. Did you perform revision arthroplasty in the first weeks after the lockdown?

- Yes

- No

- No, I have done very few even before the corona pandemic

13. Can you do the same number of procedures on an OR day compared to precorona times?

- Yes, the same number of procedures per OR day

- No, due to the less available OR time per day

- No, anesthesia and patient changes take more time

- I can plan more procedures compared to precorona times

14. Has the admission time for total knee arthroplasty decreased?

- Yes

- No

15. If the admission time for TKA has decreased, please complete below (number of nights). (If you answered no to the previous question, please leave this question open)

- # admission nights precorona times

- # admission nights currently

- Not applicable

16. Has extra space been created by the hospital to operate outside normal hours? (e.g. later in the evening or at the weekend)

- Yes

- No

17. How many patients did you see (on average) on the outpatient clinic before the corona crisis per week?

18. How many patients could you see on the outpatient clinic the first week after the measures were released (beginning of May)?

19. How many patients can you see now (as of 8 June) per week on the outpatient clinic?

20. Have you taken or are you going to take measures to eliminate waiting lists? (several options possible)

- No, I just continue working as a pre-corona

- I extend to the evening consultations

- I extend to weekend work

- I extend to the a private clinic

- I'm going take les holiday days

21. Is there enough room in the hospital to give all staff members room to perform outpatient clinics?

22. If there is not enough room for all staff members, how do you deal with this? (several options possible)

- More evening sessions

- More activities in private clinic setting

- More weekend activities

- Others

23. How large is the demand for outpatient visits?

- Larger demand compared to precorona times

- Same demand compared to precorona times

- There is less demand compared to precorona times (patients seem to be reluctant)

24. Has your waiting time for outpatient visits increased?

- Yes

- No

25. Has your waiting time (as of 8 June 2020) for surgery increased?

- Yes

- No

26. If your OR waiting time has increased, please complete below. (if you answered no to the previous question, please leave this question open)

- # weeks pre corona times

- Now, #

27. What measures are taken at your department for outpatient visits? (several options possible)

- No measures

- Waiting room according to social-distance rules

- Patients come alone as much as possible

- Patients should wear a mouth mask

- Patients need to disinfect hands

- Others

28. Do you still use teleconsultations?

- Yes, and I would like to continue to do so in the future

- Yes, but once everything is back to normal I will stop using it

- No

29. Were you or the nursing staff tested for COVID-19 at the resumption of elective activities (PCR or antibody) (multiple options possible)

- Surgeon

- Scrub nurses

- Ward nurses

- Secretary employees

- Not routinely

30. Have you been infected yourself / had symptoms of COVID 19 infection?

- Yes

- No

31. Are patients tested for COVID-19 if it concerns same day discharge procedures?

- Yes

- No

32. What measures are taken by you or nursing staff before elective surgery? (several options possible)

- Preoperative symptoms questionnaires

- Swab, PCR (<72h prior to surgery)

- Swab (PCR) and antibody test (<72h prior to surgery)

- Same day vital signs monitoring

- Blood test (inflammatory parameters)

- Questionnaires on same day of surgery

- Others

33. Have you made surgical technical adjustments as a result of COVID-19? (several options possible)

- No

- More usage of tourniquet

- Less usage of electrocautery (or more suction on electrocautery)

- Less use of pulse lavage

- Others

34. What specific personal measures do you take during surgery? (several options possible)

- If the patient has tested negative, I am wearing a surgical mouth mask

- I always try to wear at least an FFP2 mask

- I am trying to wear protective glasses more often

- I am not taking any special precautions

- Others

35. Has your preference for anaesthesia changed?

- No, I used mainly loco-regional anaesthesia anyway

- Yes, I use more loco-regional anesthesia now

- No, I prefer general anaesthesia

36. Are other anaesthesiological or general measures being taken? (several options possible)

- Extra time between procedures to ventilate the OR

- Restriction of staff in the OR at the time of induction of general anaesthesia

- Preference for locoregional anaesthesia

- Ventilation of the OR always on septic surgery

- Others
