# Supplementary material for: The long road to recovery: at six months since the first COVID-19 wave, elective orthopedic care has still not fully recovered in Belgium
Source: J Exp Orthop. 2020 Dec 21;7:99. doi: 10.1186/s40634-020-00316-9 (PMC7752098; doi:10.1186/s40634-020-00316-9)
Supplement: Supplementary file 3 — Additional file 3. Overview of survey 3. [file 40634_2020_316_MOESM3_ESM.docx]

1. In which province are you working?

- Antwerp
- Limburg
- East Flanders
- West Flanders
- Flemish Brabant

2. Where is your main working activity?

- University Hospital

- Non-University Hospital

3. How many years of experience do you have as an orthopedic surgeon?

- <5 years

- 5-10 years

- 10-20 years

- >20 years

4. How many elective surgery procedures have you done in the last week?

5. What percentage of elective procedures did you perform compared to precorona times?

- <25%

- 25%

- 50%

- 75%

- 90%

- 100%

- >100%

6. How many outpatient visits did you conduct the last week?

7. What percentage of outpatient visits did you perform compared to precorona times?

- <25%

- 25%

- 50%

- 75%

- 90%

- 100%

- >100%

8. How much OR time do you have compared to precorona times?

- <25%

- 25%

- 50%

- 75%

- 90%

- 100%

- >100%

9. How long was your OR waiting time just prior to the corona crisis? (weeks)

10. How long is your OR waiting time now? (weeks)

11. How many teleconsultations did you conduct the last week?

12. How large is the demand for outpatient visits?

- Larger demand compared to before the corona crisis

- Same demand compared to before the corona crisis

- There is less demand compared to before the corona crisis (patients seem to be reluctant)

13. How many elective procedures have you had to cancel/postpone since the restart because of preoperative positive COVID test?

14. Are you aware of patients who have contracted a COVID-19 infection during admission for elective orthopedic surgery since the restart of elective care?

- Yes, #

- No

15. Are you aware of any patients who have contracted a COVID-19 infection which can be traced back to an outpatient visit since the restart of elective care?

16. Have you had a proven COVID-19 infection?

17. If so, can it be linked to hospital activities?
